# Functional genomics in stroke: current and future applications of iPSCs and gene editing to dissect the function of risk variants

**DOI:** 10.1186/s12872-023-03227-6

**Published:** 2023-04-29

**Authors:** Alessandra Granata

**Affiliations:** Department of Clinical Neurosciences, Victor Phillip Dahdaleh Heart & Lung Research Institute, Papworth Road, Cambridge Biomedical Campus, University of Cambridge, Cambridge, CB2 0BB UK

**Keywords:** Induced pluripotent stem cells, Stroke, Small vessel disease, Genetic risk variant, Genome editing, Disease modeling

## Abstract

Stroke is an important disease with unmet clinical need. To uncover novel paths for treatment, it is of critical importance to develop relevant laboratory models that may help to shed light on the pathophysiological mechanisms of stroke. Induced pluripotent stem cells (iPSCs) technology has enormous potential to advance our knowledge into stroke by creating novel human models for research and therapeutic testing. iPSCs models generated from patients with specific stroke types and specific genetic predisposition in combination with other state of art technologies including genome editing, multi-omics, 3D system, libraries screening, offer the opportunity to investigate disease-related pathways and identify potential novel therapeutic targets that can then be tested in these models. Thus, iPSCs offer an unprecedented opportunity to make rapid progress in the field of stroke and vascular dementia research leading to clinical translation. This review paper summarizes some of the key areas in which patient-derived iPSCs technology has been applied to disease modelling and discusses the ongoing challenges and the future directions for the application of this technology in the field of stroke research.

## Background

**Fig. 1:**
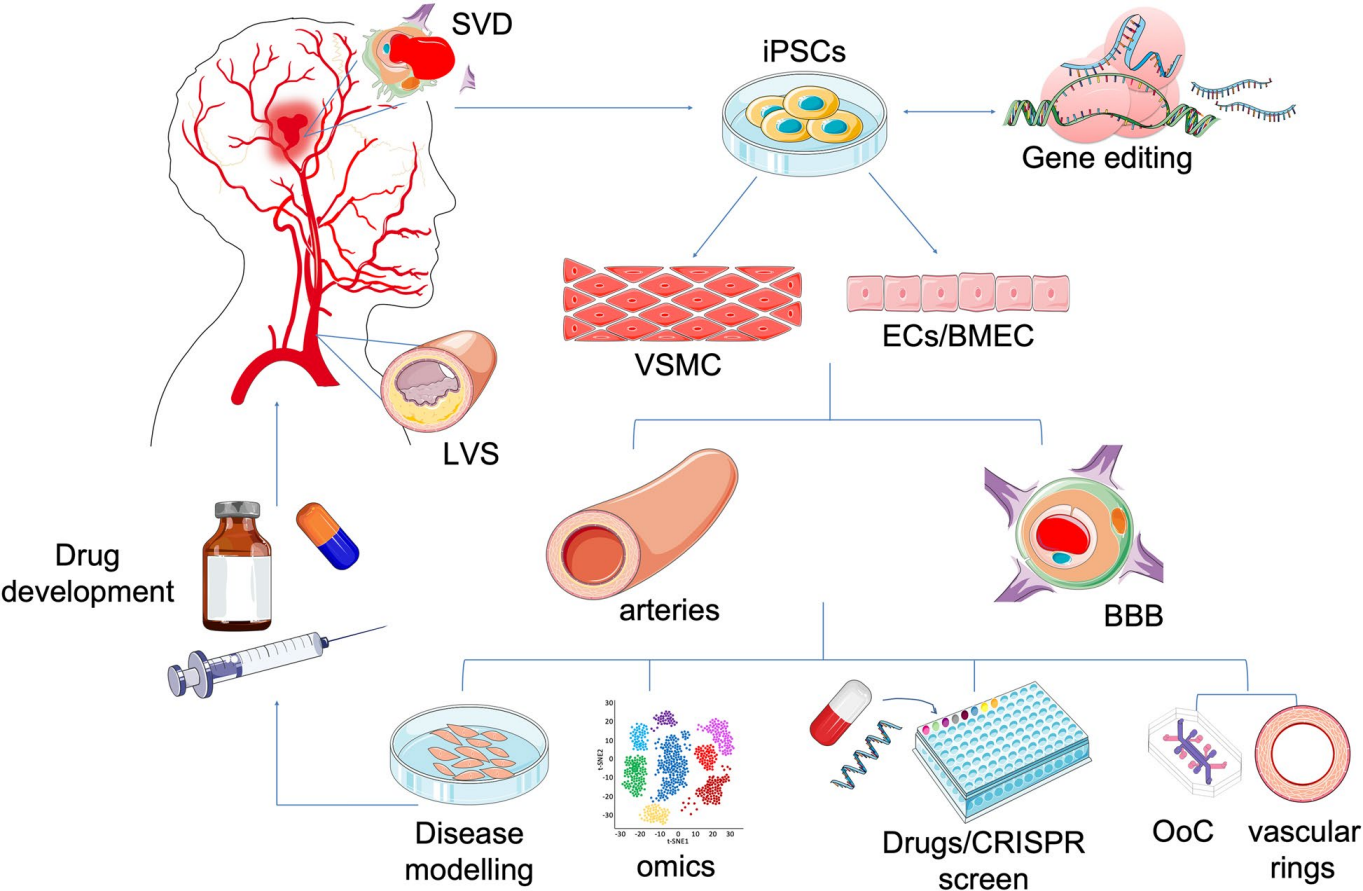
Overview of the current and future applications of human iPSCs technology in the research for large-vessels stroke (LVS) and small vessel diseases (SVD) to develop relevant vascular models, made of vascular smooth muscle cells (VSMC), endothelial cells (ECs) and brain microvascular endothelial cells (BMEC) to model the disease ‘in a dish’ and identify new therapeutic targets for future treatments of stroke. (BBB = blood brain barrier; OoC = Organ-on-Chip). Parts of the figure were drawn by using pictures from Servier Medical Art (http://smart.servier.com/), licensed under a Creative Commons Attribution 3.0 Unported License (https://creativecommons.org/licenses/by/3.0/)

Stroke is the fourth leading cause of death in the UK and the second in the world [1]. It is also the leading cause of long-term disability and vascular dementia, which is the second most common form of neurological condition after Alzheimer’s disease [2]. Despite the size of the health burden it causes, there has been little progress in understanding the underlying risks of stroke and developing new treatments. Conventional cardiovascular risk factors, including hypertension, smoking, diabetes mellitus and hyperlipidaemia are important in stroke risk [3]. However, common risk factors fail to account for all stroke risk, as a proportion (~ 50%) of the risk of stroke remain unexplained [4]. The focus of this review is to describe the current and future applications of patient-derived induced pluripotent stem cells (iPSC) technology into the research of stroke and cerebral small vessel diseases, a common cause of vascular dementia. Here, I will summarize findings in stroke genetics and address how the use of iPSC technology could help us to further investigate the pathological mechanisms driven by these genetic risk factors for stroke. I will explore the potential of the application of patient-derived iPSC in combination with genetic manipulation for disease modelling and drugs screening for the research into stroke and small vessel diseases as well as describing current limitations and challenges (Fig. 1).

### Subtypes and current treatments

There are two main forms of stroke: ischemic stroke, caused by a blood clot in the brain, and haemorrhagic stroke, caused by a bleed in the brain. Most stroke cases (~ 80%) are ischemic and can be further divided in subtypes, including cardioembolic, atherosclerotic and lacunar [3]. Of all ischemic strokes, 20% are related to large-artery stroke (LAS) caused by atherosclerotic stenosis of the major intracranial arteries, and 20–30% to cardioembolic stroke (CES). Lacunar stroke is a marker of cerebral Small Vessel Disease (SVD) and accounts for 25% of ischaemic stroke. SVD has been the focus of recent investigations because of the strong genetic components and lack of mechanistic understanding despite it has been recognised as the most common pathology underlying vascular dementia and vascular cognitive impairment [5]. The term SVD refers to several clinical and radiological features which describe disease of the small perforating arteries, arterioles, capillaries that are in the brain parenchyma supplying the white and deep grey nuclei of the brain [6].

SVD accounts for up to a fifth of all strokes, typically causing ischaemic lacunar strokes, but it is also now recognised as an important contributor to deep intracerebral haemorrhage. SVD can be further divided into two categories: a sporadic common form and rare monogenic forms, which account for 1% of all strokes overall. Importantly, both rare and common forms of SVD share genes and correspondent biological pathways [7, 8].

The primary prevention of stroke includes lifestyle modification, such as dietary changes and smoking cessation, and treating medical conditions, such as atherosclerotic disease, hypertension, and diabetes. Ischaemic stroke is treated with drugs that dissolve blood clots (thrombolytics), reduce blood clotting (anticoagulants) and lower blood pressure (antihypertensives). Haemorrhagic strokes may require surgery to remove blood and repair burst blood vessels. However, preventative therapies are slow to emerge because of the lack of understanding of the underlying biological mechanisms leading to stroke. For instance, advances in neuroimaging suggest that damage to the blood–brain-barrier (BBB) of the brain penetrating capillaries, is an early mechanism in SVD, however the cascade of events leading to BBB leakage still needs to be elucidated [9].

### Genetic risk factors of stroke

In addition to common risk factors, research has focused on genetic factors influencing the risk of atherosclerosis and blood clotting. Hereditary factors are important risk factors for stroke and could contribute to stroke risk through several potential mechanisms [10, 11].

Firstly, there are specific single-gene disorders that may cause rare, hereditary disorders for which stroke is a primary manifestation. For instance, the most common form of monogenic SVD is cerebral autosomal dominant arteriopathy with subcortical infarcts and leukoencephalopathy (CADASIL), caused by Notch Receptor 3 (*NOTCH3*) mutations and primarily affecting middle-aged individuals, causing recurrent strokes, mood disorders, and cognitive impairment leading to dementia and disability [12]. Genetic causes of conventional stroke risk factors, such as atrial fibrillation, diabetes mellitus and hypertension, are also associated with risk of stroke [13]. Lastly, some common variants of genetic polymorphisms have been associated with stroke risk (e.g., variants on chromosome 9p21) and emerging evidence from genetic studies could help to distinguish stroke subtypes and develop personalised medicine [14].

Recent genome-wide association studies (GWAS) suggest that 60 different genetic variants influence the risks of different subtypes of stroke, and therefore might functionally contribute to underlying pathogenesis [15, 16]. Among the risk variants identified by GWAS, a common single nucleotide polymorphism (SNP) rs2107595 in the histone deacetylase 9 (*HDAC9)* gene is the strongest genetic risk for the large-vessel stroke (LAS) subtype only [17].

The *HDAC9* risk variant was also found to be associated with carotid intima-media thickness and coronary artery diseases, suggesting a role for *HDAC9* in promoting atherosclerotic pathogenesis [18]. Other stroke subtypes specific variants include SNPs in both the paired-like Homeodomain Transcription Factor 2 (*PITX2*) and the Zinc Finger Homeobox Protein 3 (*ZFHX3*), both of which were initially associated with atrial fibrillation, a well-recognised risk factor for stroke, and then found to be associated with the cardioembolic stroke subtype [19]. Whereas Forkhead transcription factor 2 (*FOXF2)* gene was found to be associated with both the risk of all types of strokes and with the white matter hyperintensity burden, a marker of SVD [20]. Determining the molecular functions of these loci could be instrumental in identifying new druggable targets and developing therapeutic approaches for specific stroke subtypes.

### Cerebral vessels and vascular cells affected in stroke

#### Large-artery stroke

One of the most common causes of stroke is atheroma of the neck and head large arteries, which contain two primary major cell types: endothelial cells (EC) and vascular smooth muscle cells (VSMC) [21, 22]. Both EC and VSMC play an essential role in sustaining vascular homeostasis, and both cells type dysfunctions and aberrant interactions can contribute to the pathogenesis of atherosclerosis [23–25]. For instance, VSMC contribute to all-stages of atherosclerosis: dysregulated VSMC proliferation contributes to early-stage plaque formation and extracellular matrix (ECM) deposition to form the fibrous cap; VSMC phenotypic switching can also promote aberrant inflammation, cell senesce and ultimately plaque rupture in advance lesions [26]. Equally, endothelial dysfunction, characterised by impaired nitric-oxide (NO)-dependent vasodilatation, enhanced oxidative stress, altered metabolism, endothelial-to-mesenchymal transition and inflammation is a recognised driver of atherosclerosis [24].

#### Small vessel disease

The large arteries of the neck merge into the circle of Willis, a polygon of interconnected vessels at the base of the brain, which give rises to intercerebral arteries, and pial arteries distributed along the surface of the brain.

From the pial arteries, the emerging vascular network, which penetrates the brain parenchyma perpendicular to the brain surface, includes arteries and arterioles and capillaries and is found to be disrupted in cerebral SVD [27, 28]. In SVD brains, a combination of imaging studies with cerebral blood flow and metabolism measurements using positron emission tomography releveled a series of changes in white matter and subcortical grey matter, including recent small subcortical infarct, lacunes, white matter hyperintensities, enlarged perivascular spaces, microbleeds as well as blood–brain-barrier (BBB) impairment, eventually leading to brain atrophy [29–31].

The BBB is a unique functional structure found at the level of brain arterioles and capillaries, which is formed by brain microvascular endothelial cells (BMEC) connected by extensive tight junctions, with limited trans- and para-cellular transport, compared to endothelial barriers elsewhere in the body [32]. At the capillaries level, VSMC are replaced by pericytes, which are abundant in brain vessels and are involved in the development and maintenance of the BBB [33, 34]. In addition to pericytes, cerebral capillaries are surrounded by astrocytic end-feet, which cover more than 90% of capillaries and contribute to the BBB regulation [35]. In addition to the cellular component, there is an acellular part called basal lamina, a thin extracellular matrix layer which support BBB integrity and cell–cell communication. These three cell types together with neurons and the basal lamina, are the main components of the neurovascular unit (NVU), which is believed to be an important key player in stroke pathology [36]. Growing evidence indicates a significant role for the NVU, including both cellular dysfunction and matrix abnormalities, in the breakdown of the BBB, leading to increased permeability affecting the cerebral microvascular in the pathophysiology of SVD and stroke [37–39].

Thus, it is of crucial importance to be able to develop experimental models for both large-vessels and small-vessels using the relevant human cell types to establish the causality of the variant-stroke subtype association and the underlying biological mechanisms.

### Experimental models

Animal models are invaluable experimental models to study basic mechanisms, disease progression and risk factors, such as environmental and dietary factors related to stroke. However, the use of animal models for fully assessing a complex polygenetic disorder like stroke is questionable and most therapeutic discoveries obtained from animal models are ineffective in human clinical trials. This is because rodent models differ from humans in term of lifespan, brain size, white and grey matter volume ratio and size and morphology of deep penetrating arteries. For comprehensive details on these models, the reader is further referred to in-depth reviews [40–42]. One example is the transgenic mice carrying the CADASIL-causing Notch-3 R169C mutation, which is found to develop granular osmiophilic deposits (GOM) in brain vessels characteristic of CADASIL patients, progressive white matter damage, and reduced cerebral blood flow [43]. However, CADASIL mice have a normal lifespan, and no stroke lesions.

Thus, the need to develop novel robust human models, which will complement animal models for stroke and provide a system in which disease-causal cells can be investigated and manipulated, leading the way to functional genomics and multilevel omics of stroke.

## Current and potential applications of human iPSCs in functional genomics

The emergence of induced pluripotent stem cell (iPSCs) technology has had a tremendous impact in the field of disease modelling since its discovery in 2007 and has contributed to revolutionize the way we study complex diseases using human patient cells [44]. There is therefore great potential for patient-derived iPSCs technology in combination with genome editing techniques to maximize the value of functional genomic data and accelerate its translation into stroke clinic.

In recent years, several protocols have been developed with the aim to differentiate human iPSCs into cells that closely resemble the primary human cells of the cerebrovasculature, including BMEC, VSMC and pericytes [45]. Thus, using patients-derived iPSC to generate vascular cells could provide a powerful and reliable model system for studying stroke biology, disease modeling and drug screening.

Moreover, combing patient-derived iPSC technology with genome editing technique allows the generation of isogenic cell lines that differ in single genetic changes for causal modeling of candidate variants, offering a new tool to investigate the genotype–phenotype relationship involved in stroke pathogenesis. In addition, the generation of clinically relevant numbers of vascular cells from patient-derived iPSC holds great promise as therapeutic agents for tissue repair and regeneration post stroke [46].

### Disease modeling

Patient-derived iPSCs have been already successfully applied to the research into cardiovascular diseases [47]. There are several examples of generation and application of patients’ iPSC-derived vascular cell types for modeling of cardiovascular diseases including cardiomyocytes (e.g. cardiomyopathy-long QT syndrome (LQTS)), endothelial cells (e.g. pulmonary arterial hypertension and Fabry disease), vascular smooth muscle cells (i.e., aortic aneurysm-Marfan syndrome), macrophages (e.g. Gaucher disease) and megakaryocytes (e.g. platelet disorders) as well as for cardiac cell therapy [48–54].

In recent years, a number of patients’ iPSCs-derived models have been developed for CADASIL, the most common form of genetic SVD [55]. In one of these studies, CADASIL patient’s iPSCs were differentiated into VSMC, which show gene expression and functional changes associated with the disease phenotypes, including activation of NOTCH and nuclear factor kappa B (NF-κB) pathways, increased proliferation rate and altered cytoskeletal features [56]. In comparison, these defects were not seen in EC derived from the patient’s iPSCs, suggesting a cell type specific penetrance of these molecular phenotypes. In another work, iPSCs generated from two CADASIL patients, were subsequently differentiated into pericyte-like cells, showing decreased PDGFRβ levels and reduced VEGF secretion, which might result in impaired stabilisation of capillary structures [57]. On the contrary, Yamamoto et al. described an increased level of PDGFRβ in iPSCs-derived VSMC from three CADASIL patients, which remarkably recapitulate NOTCH3 extracellular domain accumulation as seen in patient’s biopsies [58, 59]. Importantly, treatment with NOTCH inhibitors or specific siRNA appear to be beneficial in alleviating the phenotype in the different iPSC-derived models, indicating a potential therapeutic intervention strategy for CADASIL. However, discrepancies across studies, which might be the consequence of differences in differentiation protocols, should be addressed to improve the reliability of phenotype comparison between iPSCs models.

Our group has recently developed a patient’s iPSCs-derived VSMC model for the stroke risk variant rs2107595 in the *HDAC9* gene, which has the strongest association with LAS identified to date and has been linked to advanced carotid atherosclerosis [60]. We found that *HDAC9* is associated with increased cell death and inflammatory response upon stimulation with the pro-inflammatory cytokine Tumour Necrosis Factor alpha (TNF-α) in VSMC, which are important cells for atherogenic process and plaque stability. This is the first example of a functional genomic study for a risk variant associated with a stroke subtype using human iPSCs and opens the door to investigate further candidate genes.

At present, a wide range of research tools are available to maximize the efficacy of patient-derived iPSC technology and advance the research into stroke and SVD (Table 1), which will be discussed here.

**Table 1:** Summary of the iPSCs applications

iPSCs applications	Methods	Value	References
**Genome editing**	Gene correction	Generation of isogenic controls for disease modelling	[61–63]
	Gene insertion	Generation of novel mutation	[64–66]
	Gene knockdown	Perform eQTL studies	[67]
	CRISPR-mediated gene interference (CRISPRi) or gene activation (CRISPRa)	Investigate GWAS risk variants function	[68, 69]
	CRISPR-based screening	Investigate known genetic modifiers	[70, 71]
		Identify novel therapeutic targets and biomarkers	[72, 73]
**3D cell models**	Organoids	Mimic organ functions in vitro	[74–77]
		Replicate the pathology in vitro and discover underlying pathologic mechanisms	[78, 79]
		Develop high-throughput screening platform	[80]
	Engineered tissues i.e., Vascular rings	Mimic organ functions in vitro	[81–83]
		Replicate the pathology in vitro	[84]
	Organ-On-a-Chip	Mimic organ functions	[85–87]
		Model the pathology and discover therapeutic targets/pathways	[88, 89]
		Develop platform for high-throughput screening	[90, 91]
**Multi-omics**	Genomics, Epigenomics, Transcriptomics, Proteomics, Metabolomics	Provide insights into disease mechanisms	[92, 93]
		Identify potential diagnostic biomarkers	[94]
**Drug screening**	Phenotypic screening of drug libraries	Uncover underlying therapeutic targets/pathways	[80]
		Test effectiveness of candidate drugs	[60, 95]
	Novel compounds	Test drug toxicity	[96]
		Test drug permeability	[90, 97]

### Genome editing technology

The use of genome editing tools in combination with both patient-derived and healthy donor (wild-type; WT) iPSC technology offers a powerful approach to validate GWAS results, by reducing the disease confounders and isolating the effect of the risk variant in a relevant model, which could be applied to determine the functions of GWAS-identified coding and noncoding variants in stroke [98]. Genome editing technologies, such as zinc finger nucleases, transcription activator-like effector nucleases (TALEN), and clustered regularly interspaced short palindromic repeats (CRISPR) methodologies, have been applied to both WT and patient-derived iPSCs to study the effect of risk variants in a range of cardiovascular disease models, including BLC2-associated athanogene 3 (*BAG3*) gene in dilated cardiomyopathies and Notch Receptor 1 (*NOTCH1*) gene in congenital valvular disorder [61, 64, 99].

Genome editing strategies for functional genomics include allele substitution to ‘correct’ the risk variant while leaving the patients-derived iPSCs otherwise unchanged to create isogenic controls (e.g. correction of *SCN5A* mutations and cardiac channelopathy variant in LQTS in patients’ iPSC-derived cardiomyocytes) [62, 63]. Alternatively, editing can be used to introduce a variant into WT iPSCs, when patient’s cells are not available, to create a disease line. For instance, chronic kidney disease was modelled by inserting the nephropathy associated *APOL1* risk variant into WT iPSCs-derived kidney organoids, without the need to recruit patients with this mutation [65]. In another study, introduction of homozygous *APOE4* alleles in WT iPSC showed increased susceptibility to Alzheimer’s disease [66].

Besides assessing the effects of pathogenic variants on disease phenotypes, the gene-editing toolbox have been successfully used to dissect molecular mechanisms in in vitro models by 1) relating gene function to phenotypes using gene knockout or knockdown approaches; 2) relating coding variants to protein function, and non-coding variants to gene expression by expression quantitative trait loci (eQTL) studies [67]. For instance, the regulatory effect of a coronary artery disease-associated genomic locus on Endothelin-1 expression was assessed in patients’ iPSCs-derived EC and VSMC by introducing indel nearby the causal variant to disrupt the core regulatory sequence [68]. Equally, CRISPR-mediated gene interference (CRISPRi) or gene activation (CRISPRa) have been used to repress or enhance the activity of local cis-regulatory elements to investigate the functions of noncoding variants [100]. Seminal work from Schrode et al., has shown how simultaneous modulation of endogenous gene expression at loci containing several schizophrenia-associated SNPs through CRISPRi/a allows us to unravel the synergic contribution of common risk variants to complex genetic disorder [69].

In addition, high-throughput CRISPR-based screen can be employed for high-throughput interrogation of known genetic modifiers. For instance, a recent study has used CRISPRi screen to investigate thousands of noncoding variants at the TNF-α-induced protein 3 (*TNFAIP3*) region, a genetic locus associated to multiple autoimmune diseases, to pinpoint the disease causal variants [70]. CRISPR-mediated approaches have also been used to study the effects of genetic variants on the function of putative enhancers via saturation mutagenesis genome editing. For instance, GWAS studies have identified an association between ∼10-kb enhancer of BAF Chromatin Remodeling Complex Subunit (*BCL11A)* and foetal haemoglobin levels, which could have therapeutic potential for diseases such as sickle-cell anaemia and β-thalassemia [71]. To functionally fine-map this region, a variant-aware saturating mutagenesis was performed using multiple nucleases with different Protospacer Adjacent Motif (PAM) sequences and genome modification was assessed by cells sorting for foetal haemoglobin expression. These studies were performed in immortalised and primary cell lines, however the use of iPSCs would be advantageous because provides a powerful mean to investigate the effect of variant manipulation in disease relevant cell types.

This CRISPR-mediated interference/activation technology is especially relevant for stroke research since many stroke-associated risk variants are in a non-coding site which could affect the expression of neighboring genes (i.e., rs2107595 SNP in the *HDAC9* gene) as well as being involved in the long-range regulation of gene expression in a tissue-specific way. Furthermore, CRISPR-based screen could be used to screen open chromatin regions, transcription factors or histone marks associated, and even the whole genome [101]. This approach has been successfully applied to iPSC-derived neurons to identify mechanisms of selective vulnerability in neurodegenerative diseases [72]. Genome-wide CRISPR screen led to the identification of a druggable suppressor of sarcoma cancer stem cells, the Krueppel-like factor 11 (KLF11), which if pharmacological activated in synergy with chemotherapy could be improve the success rate for osteosarcoma treatment [73]. This supports the idea of applying genome-wide CRISPR screen to iPSCs-derived vascular models of stroke to identify novel therapeutic pathways and diagnostic biomarkers.

### 3D iPSC models

Most iPSCs disease modelling studies use the conventional 2-dimensional (2D) monolayer culture systems, which offer a rapid method to model cell deficit. However, the 2D culture system lacks cell–cell contacts and tissue- organ-specific extracellular matrix mechanisms, which are crucial to replicate disease pathophysiology. To meet these needs, recent efforts have been directed to develop 3-dimensional (3D) culture systems.

3D models include: 1) self-organized organoids, which adopt matrices mimicking the native ECM, including matrigel, successfully utilized to support intestinal organoids [74]and fibrin hydrogel to encapsulate liver organoids [75]. 2) Engineered tissues, such as heart tissue and vascular rings, which offer a controlled environment enabling tissue maturation, while providing cell interactions and functional readouts [81–83]. 3) Organ-On-a-Chip (OOC) which combines engineered single or multi-tissue units with microfluidic flow, to recapitulate complex physiological function, such as contractile function in the heart by promoting cardiomyocytes maturation and filtration in the kidney by co-culturing iPSC-derived podocytes with endothelial cells to mimic cell–cell interactions [85, 86].

Organoids are 3D cell self-organised masses that recapitulate some level of tissue or organ structure and function. A range of WT and patients’ iPSC-derived organoids resembling different organs, including brain and heart have been successfully developed to model human diseases. Remarkably, Skylar-Scott et al., have recently introduced a biomanufacturing method that combine densely cellular matrices with patients’ iPSC-derived organoids to produce perusable organ-specific tissues of arbitrary volume and shape at therapeutic scale [76]. Lewis-Israeli et al. was successful in applying this technology to generate a developmentally relevant human heart organoid by self-assembly using WT iPSCs to study congenital heart defects [78]. Furthermore, work from Wimmer et al., explored how a self-organising 3D human blood vessel organoid from WT iPSCs can be perfused to mimic the features of human microvasculature to model diabetic vasculopathy [79].

In recent years, the research into neurodegenerative diseases has also greatly benefitted from the progress in cerebral organoids technology with the generation of ‘mini-brains’ which recapitulate both the multicellular and structural aspects of the human brain to explore both developmental and pathologic processes [77, 102]. However, the lack of a vascular network limits the differentiation and maintenance of these organoids due to the limited supply of nutrients and oxygen to the core cells. Thus, the incorporation of a vascular network mimicking the BBB function, would not only contribute to the development of the mini-brain but also offer disease-modelling opportunities for neurodegenerative diseases, SVD and stroke [103].

Patients’ iPSCs-based stroke models could also benefit from the application of tissue-engineered vascular rings, which have been previously use to assess contractility in an in vitro model of thoracic aortic aneurysm [84]. These self-assembled vascular rings made of iPSC derived VSMC, change their circumference in response to vasoconstrictors, thus providing an effective tool to evaluate the effect of vessel contractility (as seen e.g. in aortic stenosis) in an in vitro model of stroke.

Moreover, OOC is an emerging technology which uses microfluidic devices of engineered biomaterials to mimic the native extracellular matrix, introduce the flow-induced shear-stress and support the seeding of different cell types to build engineered tissue [87]. Advances in microfluidic technology have led to the generation of several BBB-on-chip devices, which could facilitate the study of stroke and SVD as done before for Alzheimer’s disease [88] and brain tumours [89]. Recent studies support the application of OOC as platform for drug-screening and targeted delivery by enhancing the chip performance with exposure to hypoxia [90] and by building high-content assay platform suitable for compound screen [91].

In vitro BBB models are largely based on the use of primary vascular cells or immortalised cell lines, for testing drug efficiency and permeability through the barrier. Primary cells have the benefit that they keep their own phenotype and establish good physical properties, including high expression of tight junction proteins, which improves barrier tightness, often measured as transendothelial electrical resistance (TEER) [104]. However, primary cell isolation and purification is time consuming, and their application is limited by the fact that the cells lose phenotypic identity with increased passage number. Immortalised cell lines are well established, highly proliferative, and able to maintain a constant phenotype during passaging, but they struggle to achieve physiological TEER making them ineffective for functional studies. Thus, the introduction of iPSCs-derived brain endothelial cells (iBMEC) holds great promise for drug screening and personalised medicine, since these cells carry the patients ‘causal genetic defects and exhibit physiologically relevant TEER for accurate permeability study of BBB models [105].

However, a recent controversy has emerged regarding the validity of iBMEC for in vitro studies [106]. Despite iBMEC having been widely adopted for their capacity to mimic physiological BBB properties, in depth characterisation combining protein analysis and transcriptomic profiling has shown that iBMEC lack canonical endothelial cell transcriptional identity and conversely expressed some epithelial markers, such as epithelial cellular adhesion molecule (EPCAM), at both RNA and protein levels, therefore making them unsuitable for in vitro disease modelling [107]. Thus, it is essential that improvements are made to the differentiation protocols and culture processes to produce iBMEC with similar transcription signature to the in vivo counterpart. For instance, multi-cellular 3D co-culture microfluidic models, including iPSC-pericytes and astrocytes as well as introducing laminar flow, would be beneficial in promoting BMEC maturity and enhancing brain barrier properties [97]. Ultimately, it is of critical importance to validate any new differentiation protocol for iBMEC by multi-omics approach to develop a faithful and reproducible in vitro BBB model for accurate disease modelling applications.

### Multi-Omics

GWAs to date have identified strong associations with specific stroke subtypes, but their mechanisms are still mainly unknown. Now the integration of multi-omics approaches including epigenomics, transcriptomics, proteomics, and metabolomics offers a tremendous opportunity to advance our knowledge into the process of stroke and its mechanisms [108].

Omics data can be useful to identify changes associated with diseases, however, cannot identify causal genes, which would require genetic perturbation (knockdown/knockout, overexpression) to demonstrate causality (i.e., necessity and sufficiency). Thus, target validation in patient-derived iPSCs could contribute to the identification of therapeutic targets and biomarkers that could be clinically used as shown for cardiac hypertrophy [92].

Several human consortia produced a large body of genomics, transcriptomics and epigenomics data in multiple tissues, which could provide important and unique insights into complex diseases. For instance, GTEx (http://www.gtexportal.org/home/) analyses epigenomic signatures and transcriptomics across human tissues to link genetic regulatory elements to traits and disease associations and has been successfully applied to cardiovascular disease (e.g. coronary artery disease and heart failure) [109, 110]. Moreover, UK Biobank collect samples for characterisation by various omics approaches to identify molecular changes that occur during cardiovascular disease (e.g. atrial fibrillation) [111]. Multi-omics technologies have been successfully applied before to stroke research using mouse and human brain samples [94, 112]. However, human omics suffer from limitations, including limited accessibility to samples, heterogeneity of cell type/composition, batches variability and budget. Combination of iPSCs models, including organoids and microfluidic chips, which have the potential to replicate the disease phenotype, with multi-omics approaches, could overcome these limitations and offer a platform for future studies into disease progression to identify potential causative changes [113]. In recent years, omics technologies have significantly contributed to uncover the biological heterogeneity of Amyotrophic lateral sclerosis (ALS) [114]. Recent work has combined iPSCs-derived motor neurons generated from different patients with a common ALS mutation with omics datasets to identify known and novel pathways, which were consistently dysregulated across all lines with the aim to include an additional 1,000 ALS-derived iPSC lines to define the molecular pathogenic signature for ALS [93]. Similar approach could be applied to characterise iPSCs-based models derived from patients with different stroke types to learn about early pathogenic events and develop new personalised treatments.

Moreover, multi-omics can be adopted to investigate and optimise iPSCs differentiation processes to obtain a reliable end-product and promote experimental reproducibility [115].

### Drug screening

The use of patients’ iPSCs in stroke research can not only contribute to unveil the link between genotype and phenotype, improve the understanding of the biological processes and advance disease modelling, but it can also represent a favourable model for drug screenings and for predicting the effectiveness of drug candidates, as well as their pharmacology and toxicity in humans [96, 116, 117]. Patient-derived iPSCs offers the opportunity to generate disease-specific vascular cells, i.e. EC, VSMC and cardiomyocytes, which mirror the molecular and cellular phenotype found in patients, in an unlimited amount at lower cost, providing a valuable tool for phenotypic screening. For instance Gu et al., combined functional high-throughput drug screening with pulmonary arterial hypertension patient iPSCs-derived EC and VSMC to identify compounds that improve function across all cell lines and elucidate mechanisms of action [80].

OOC are a promising approach in the pharmaceutical field, which if combined with iPSCs technology, could lead to improved target identification and validation. In this context, BBB-on-chip models are critically important for developing and delivering drugs to the brain and enormous research efforts have been spent to develop realistic BBB models to facilitate in vitro drug screening [118]. For instance, an iPSCs-based BBB-on-chip generated from Huntington’s disease patients not only confirms the increased BBB-permeability previously observed in patients but also allow us to test the selective permeability of several molecules across the barrier [97]. A similar approach could be used using iPSCs generated from patients with stroke and SVD to test drug permeability and efficacy. However, to improve the performance of these models for drug discovery application, future work should concentrate on the development of appropriate differentiation protocol for BMEC and standardization of barrier function quantification (e.g. inserting sensor for real-time monitoring of barrier permeability) [119].

Besides the development and screening of new drugs, iPSCs-based models can be used to identify already available drugs, that can be repurposed for the treatment of both common and rare diseases [120]. For example, ezogabine, an anti-epileptic drug, showed efficacy in an iPSC-derived motor neurons model of ALS, leading to the subsequent clinical trials [95]. In classical drug development assays, the results from disease models are compared to healthy controls, which differ in their genetic background and drug-response. This is an issue that can be easily overcome with the use of genome editing tools to generate the optimal isogenic controls.

In our recent study, we have set up a small-scale phenotypic screening using the iPSCs-derived VSMC model with the *HDAC9* stroke risk variant. HDAC9 belongs to the HDAC class II type of deacetylases and there are no specific inhibitors available for HDAC9. Thus, for our screening we use a set of HDAC class II inhibitors, as well as sodium valproate, an anti-epileptic drug with pan-HDAC inhibitory activity [60]. These inhibitors demonstrate positive effect on the VSMC cell survival rates, with sodium valproate been the most effective. However, since sodium valproate has significant side effects, it would be ideal to develop a specific inhibitor for HDAC9 and test its efficacy in our iPSC-derived vascular model.

### Progress and limitations

With all the great advantages of patients-derived iPSCs as human disease models, iPSCs models do have limitations [113]. One widely recognised issue is the variance in the differentiation potential of iPSC clones caused by the fact that iPSCs are generated from different donors with a variety of genetic and epigenetic profiles, reprogrammed and cultured with different methods. This will inevitably affect the reproducibility of iPSC-based disease modelling [121].

To address this issue, we could access iPSC lines generated by large-scale consortia from thousands of healthy individuals as well as patients diagnosed with selected diseases [122, 123]. The advantages of these iPSC include their systematic creation, curation, and a full set of quality control. These consortia apply rigorous characterisation procedures to examine genomic integrity to exclude lines that harbour somatic variation [124]. Moreover, these lines are accompanied by whole-exome or genome sequencing data and are subject to extensive transcriptomic and proteomic analyses.

When iPSC for the disease of interest, i.e., stroke, are unavailable from the consortia collection, the generation of lines from multiple patients and multiple sub-clones for each line should be adopted to enable the identification of line-specific outliers (e.g., somatic variation) and the validation of key results. Moreover, for known genetic variants, it is important to generate the accurate isogenic controls to remove the genetic background influence on the phenotypic effect of a mutation as previously discussed [62, 63]. When CRISPR/Cas9 editing is not applicable, multiple WT lines matched for age, sex and ethnicity and whenever possible, for the time in culture, should be used as controls.iPSC derivation and differentiation are multistep processes, which are likely to introduce small variations at each step, producing significant differences and potentially masking any biological variation of interest, especially where sample sizes are small [124]. Thus, to reduce the intra-clonal variation and improve reproducibility across different laboratories, standardization of iPSC reprogramming and differentiation should be adopted.

First, good cell culture practice must be enforced to maximize reproducibility and minimize artifacts. A second step to ensure the quality of iPSC-derived cells is a clear documentation of the protocol used to produce these cells, which need to be tested with multiple independent iPSC lines to ensure that it is robust and reproducible. When possible, highly variable cell-culture reagents (e.g., serum, protein growth factors, etc.) should be replaced with recombinant proteins or small molecules to reduce variability. Ultimately, key iPSC differentiation points as well as the terminally differentiated culture should be characterised by gene and/or protein expression studies to determine the cellular composition and homogeneity. Markers of contaminating cell fates should also be assessed as previously described for the iBMEC [107]. Moreover, the generation of more specific and efficient iPSC-differentiation protocols is benefiting from the recent development of technologies, such as single cells RNA sequencing (scRNA-seq) combined with lineage tracing and computational analysis, which are constantly improving our knowledge of developmental processes [125]. scRNA-seq also allow us to perform a quantitative and unbiased characterization of the cultured cell heterogeneity and could be. potentially introduced in the future as a quality control for each iPSC differentiation [115, 121].

Another critical problem concerns the maturation of iPSCs-derived cells, which exhibit immature characteristics comparable to foetal cell phenotypes. Particularly for modelling late-onset diseases, such as stroke, cell maturity is a critical aspect. Approaches to overcome this problem and simulate aging include chemical induction of mitochondrial stress, mechanical forces (e.g., mechanical stretching for VSMC), overexpression of progerin, a truncated version of the aging protein Lamin A and promotion of cell–cell interactions in a multi-lineages system, such as the BBB-on-chip system [126].

A further challenge for iPSC-based disease modeling is to recapitulate the in vivo environmental conditions that are not present or difficult to model under 2D conditions. 3D brain organoid-based technologies offer greater physiological relevance and the possibility to capture the complexity of events that occurs in the ischemic core and surrounding area, including excitotoxicity, production of reactive oxygen and nitrogen species, inflammation and apoptosis contributing to brain tissue damage. Constant improvement of efficient and reliable differentiation protocols of the relevant vascular cell types (i.e., BMEC) and culture techniques should be prioritised in the future to improve the quality and homogeneity of iPSCs culture. These improvements will contribute to the development of high-throughput screening with high levels of biological relevance and reproducibility, which is a critical part of drug discovery [127].

An additional issue with the adoption of genome-edited iPSCs models for phenotypic studies, is that introducing double-strand breaks into genomic DNA, however infrequent, could cleave at ‘off-target’ sites [128]. Thus, there is the possibility that the editing tools will introduce significant genomic alterations besides the desired risk variants. To mitigate any concern about genetic heterogeneity, phenotypic studies should include two or three iPSCs subclones per line, since it is unlikely that multiple clones will have the same off-target effect. Secondary editing to revert the edited gene to the original could also be adopted to confirm the phenotypic rescue. In addition, future work should focus on improving the existing editing tools to maximize on-target efficiency and minimize off-target activity.

## Conclusion

Patient-specific iPSC-based modelling is a powerful technology for the research into stroke, by furthering our understanding on how genetic variations lead to different stroke subtypes.

Although there are still obstacles, including reproducible derivation and characterisation of iPSCs-derived functional mature vascular cells, that must be overcome, we anticipate that in the future, the application of stroke iPSCs-derived 3D models, in combination with genome-editing tools, multi-omics and drug screening have the potential to accelerate the translation of GWAS to clinical impact, understand the disease molecular and cellular mechanisms to predict the clinical outcomes, and ultimately discover and validate therapeutic agents for the treatment of stroke.

## Data Availability

Not applicable.

## Abbreviations

iPSCs: Induced Pluripotent Stem Cells
SVD: Small Vessel Disease
BBB: Blood–Brain Barrier
CADASIL: Cerebral autosomal dominant arteriopathy with subcortical infarcts and leukoencephalopathy
HDAC9: Histone deacetylase 9
SNP: Single nucleotide polymorphism
GWAS: Genome-wide association studies
PITX2: Paired-like Homeodomain Transcription Factor 2
ZFHX3: Zinc Finger Homeobox Protein 3
FOXF2: Forkhead-Related transcription factor 2
VSMC: Vascular smooth muscle cells
EC: Endothelial cells
BMEC: Brain microvascular endothelial cells
LQTS: Long QT syndrome
NOTCH3: Notch Receptor 3
OOC: Organ-On-Chip
TALEN: Transcription activator-like effector nucleases
CRISPR: Clustered regularly interspaced short palindromic repeats
ALS: Amyotrophic lateral sclerosis
TNF-α: Tumour Necrosis Factor alpha
NF-κB: Nuclear factor kappa B
PDGFRβ: Platelet derived growth factor receptor beta
VEGF: Vascular endothelial growth factor
APOE: Apolipoprotein E
LAS: Large-artery stroke
CES: Cardioembolic stroke
